# Supplementary material for: Sharp-Wave Ripples Orchestrate the Induction of Synaptic Plasticity during Reactivation of Place Cell Firing Patterns in the Hippocampus
Source: Cell Rep. 2016 Feb 18;14(8):1916–29. doi: 10.1016/j.celrep.2016.01.061 (PMC4785795; doi:10.1016/j.celrep.2016.01.061)
Supplement: Document S1. Figure S1 [file mmc1.pdf]

Cell Reports, Volume *14*

Supplemental Information

Sharp-Wave Ripples Orchestrate the Induction of Synaptic Plasticity during Reactivation of Place Cell Firing Patterns in the Hippocampus

Josef H.L.P. Sadowski, Matthew W. Jones, and Jack R. Mellor

Supplementary Figure 1

a

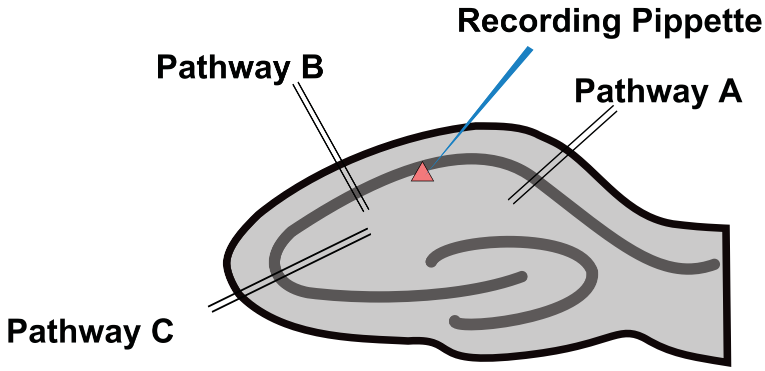

b

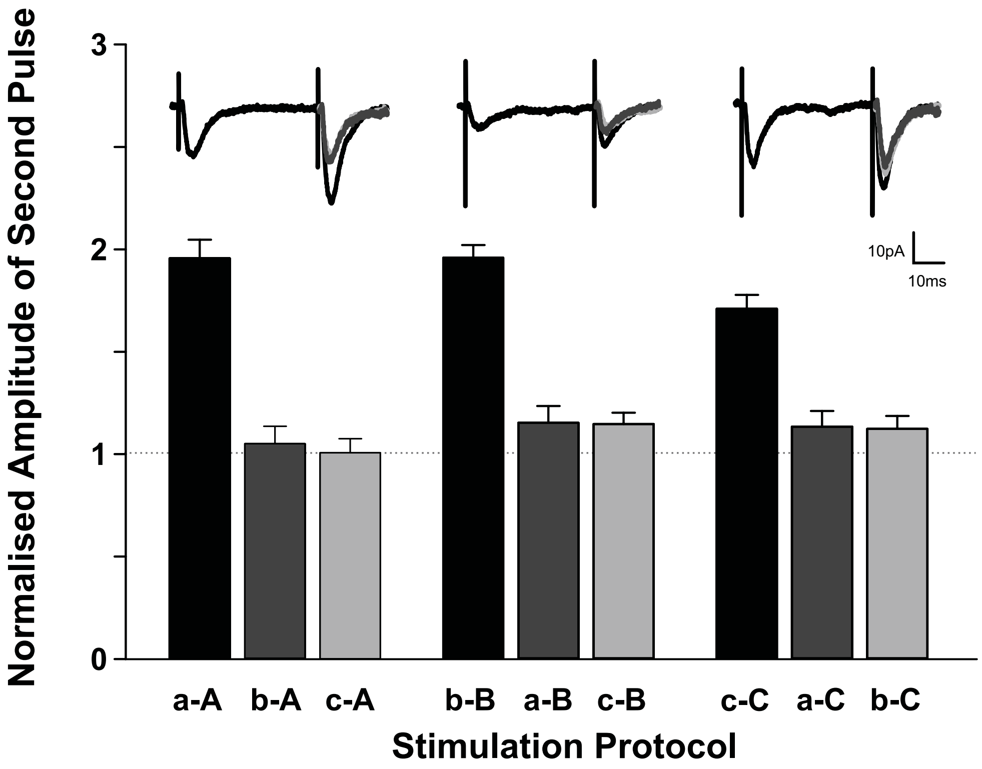

Supplemental Figure S1. Three-way Schaffer collateral pathway separation in stratum radiatum, related to Figure 2.

a) Three bipolar stimulating electrodes were positioned in stratum radiatum, activating three independent input pathways to a postsynaptic target cell in CA1.

b) EPSCs in 7 sample slices were recorded in response to a sequence of paired pulse stimuli delivered across all three pathways. Pulses were separated by 50ms. Pulses to the same pathway produced robust paired pulse facilitation where as those delivered to two opposing pathways did not. Example traces shown above.
