# Supplementary material for: Sharp-Wave Ripples Orchestrate the Induction of Synaptic Plasticity during Reactivation of Place Cell Firing Patterns in the Hippocampus
Source: Cell Rep. 2016 Feb 18;14(8):1916–29. doi: 10.1016/j.celrep.2016.01.061 (PMC4785795; doi:10.1016/j.celrep.2016.01.061)
Supplement: Document S2. Article plus Supplemental Information [file mmc2.pdf]

Cell Reports

Article

Sharp-Wave Ripples Orchestrate the Induction of Synaptic Plasticity during Reactivation of Place Cell Firing Patterns in the Hippocampus

Graphical Abstract

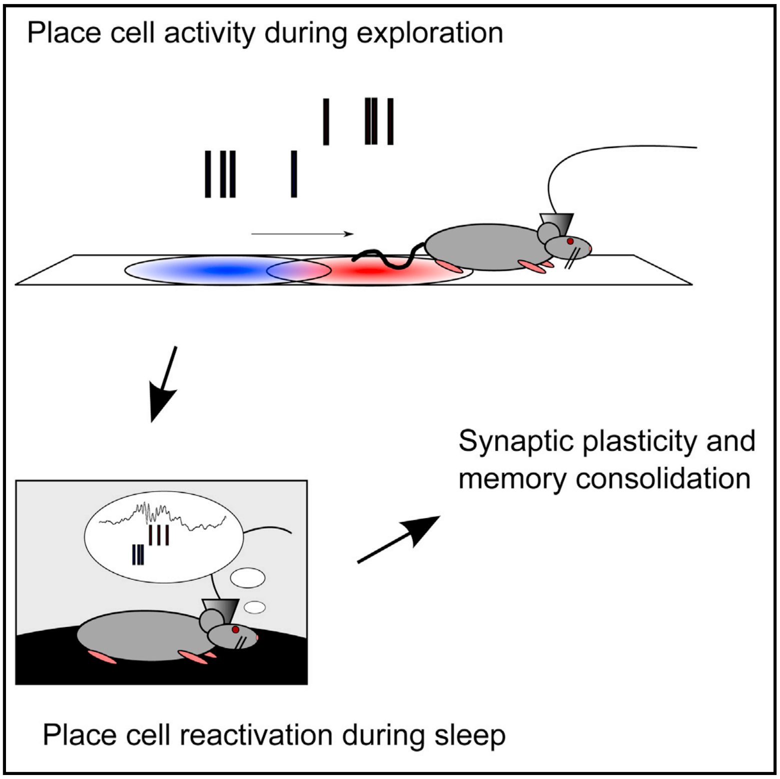

Highlights

- Reactivated place cell firing patterns can induce LTP in the hippocampus
- Sharp-wave ripples are required for the induction of LTP
- Dendritic depolarization during sharp-wave ripples is required for LTP
- The timing of place cell firing within sharp-wave ripples controls LTP induction

Authors

Josef H.L.P. Sadowski, Matthew W. Jones, Jack R. Mellor

Correspondence

matt.jones@bristol.ac.uk (M.W.J.), jack.mellor@bristol.ac.uk (J.R.M.)

In Brief

Sadowski et al. show that reactivation of neuronal firing patterns during sleep induces synaptic plasticity, providing a mechanism for the observation that memories are consolidated during sleep.

Sadowski et al., 2016, Cell Reports *14*, 1916–1929
March 1, 2016 ©2016 The Authors
http://dx.doi.org/10.1016/j.celrep.2016.01.061

OPEN ACCESS
CellPress

Cell Reports
Article

Sharp-Wave Ripples Orchestrate the Induction of Synaptic Plasticity during Reactivation of Place Cell Firing Patterns in the Hippocampus

Josef H.L.P. Sadowski,[1] Matthew W. Jones,[1,2,*] and Jack R. Mellor[1,2,*]
[1]Centre for Synaptic Plasticity, School of Physiology, Pharmacology and Neuroscience, University of Bristol, University Walk, Bristol BS8 1TD, UK
[2]Co-senior author
*Correspondence: matt.jones@bristol.ac.uk (M.W.J.), jack.mellor@bristol.ac.uk (J.R.M.)
http://dx.doi.org/10.1016/j.celrep.2016.01.061
This is an open access article under the CC BY license (http://creativecommons.org/licenses/by/4.0/).

SUMMARY

Place cell firing patterns reactivated during hippocampal sharp-wave ripples (SWRs) in rest or sleep are thought to induce synaptic plasticity and thereby promote the consolidation of recently encoded information. However, the capacity of reactivated spike trains to induce plasticity has not been directly tested. Here, we show that reactivated place cell firing patterns simultaneously recorded from CA3 and CA1 of rat dorsal hippocampus are able to induce long-term potentiation (LTP) at synapses between CA3 and CA1 cells but only if accompanied by SWR-associated synaptic activity and resulting dendritic depolarization. In addition, we show that the precise timing of coincident CA3 and CA1 place cell spikes in relation to SWR onset is critical for the induction of LTP and predictive of plasticity generated by reactivation. Our findings confirm an important role for SWRs in triggering and tuning plasticity processes that underlie memory consolidation in the hippocampus during rest or sleep.

INTRODUCTION

Synaptic plasticity is believed to mediate the encoding of memories by strengthening connectivity between co-active neurons representing constituent features of an event or environment (Hebb, 1949; Bliss and Collingridge, 1993). Recently encoded memories are liable to interference and require consolidation, a process thought to occur during rest and sleep when recently active neural ensembles are reactivated in the hippocampus (Pavlides and Winson, 1989; Wilson and McNaughton, 1994; Skaggs et al., 1996; Louie and Wilson, 2001; Lee and Wilson, 2002; Foster and Wilson, 2006; Diba and Buzsáki, 2007). During these consolidation epochs, existing hippocampal connectivity may be refined through further plasticity and consolidated engrams subsequently integrated into neocortex for longer-term storage (Frankland and Bontempi, 2005). This two-step model of memory formation therefore requires that long-term potentiation (LTP) is induced during both the encoding and consolidation stages (Buzsáki, 1989).

LTP can be induced at hippocampal synapses by intense, high-frequency stimulation of presynaptic axons, postsynaptic depolarization coupled with presynaptic stimulation (Bliss and Collingridge, 1993), or by delivering tightly synchronized pre- and postsynaptic activity (Magee and Johnston, 1997; Bi and Poo, 1998; Debanne et al., 1998; Buchanan and Mellor, 2010). The latter, also referred to as spike-timing-dependent plasticity (STDP), leads to LTP or long-term depression (LTD) according to the precise timing and temporal order of pre- and postsynaptic activity. Spike-timing-dependent LTP (STD-LTP) requires causal spiking to occur within a narrow temporal window, with a presynaptic spike followed by a postsynaptic spike within 30 ms. Anti-causal activity, whereby the postsynaptic neuron fires before the presynaptic neuron, can lead to STD-LTD. However, STDP rules are synapse- and developmental-stage-specific. For example, at mature Schaffer collateral (SC)-CA1 synapses, multiple postsynaptic spikes are required for STD-LTP (Pike et al., 1999; Buchanan and Mellor, 2007); this is important when considering the spiking requirements for STDP between co-active neurons encoding a given memory. *In vivo*, tightly correlated CA1 and CA3 pyramidal cell spiking is predicted to satisfy the requirements for STDP induction at SC-CA-1 synapses (O'Neill et al., 2010). Indeed, there are defined periods during the encoding and consolidation phases of hippocampal memory processing when CA1 and CA3 pyramidal cells are coactive and STDP may occur (Isaac et al., 2009; Bush et al., 2010a; Carr et al., 2011; Sadowski et al., 2011).

Hippocampal place cells fire in a location-dependent manner (O'Keefe and Dostrovsky, 1971), and thousands of cells in the hippocampal CA3-CA1 network are likely to have overlapping place fields, and therefore be co-activated, within a given environment (Muller et al., 1987). The firing patterns of cells with overlapping place fields may satisfy the requirements for STDP to be induced during memory encoding, for example on exploration of a novel environment (Muller et al., 1996). In fact, these firing patterns have been shown to induce LTP at SC-CA1 synapses *in vitro*, but only when cholinergic receptors are also activated in a manner that may mimic the elevated cholinergic tone observed during awake behavior (Isaac et al., 2009). This is consistent with previous evidence for induction

OPEN ACCESS
CellPress

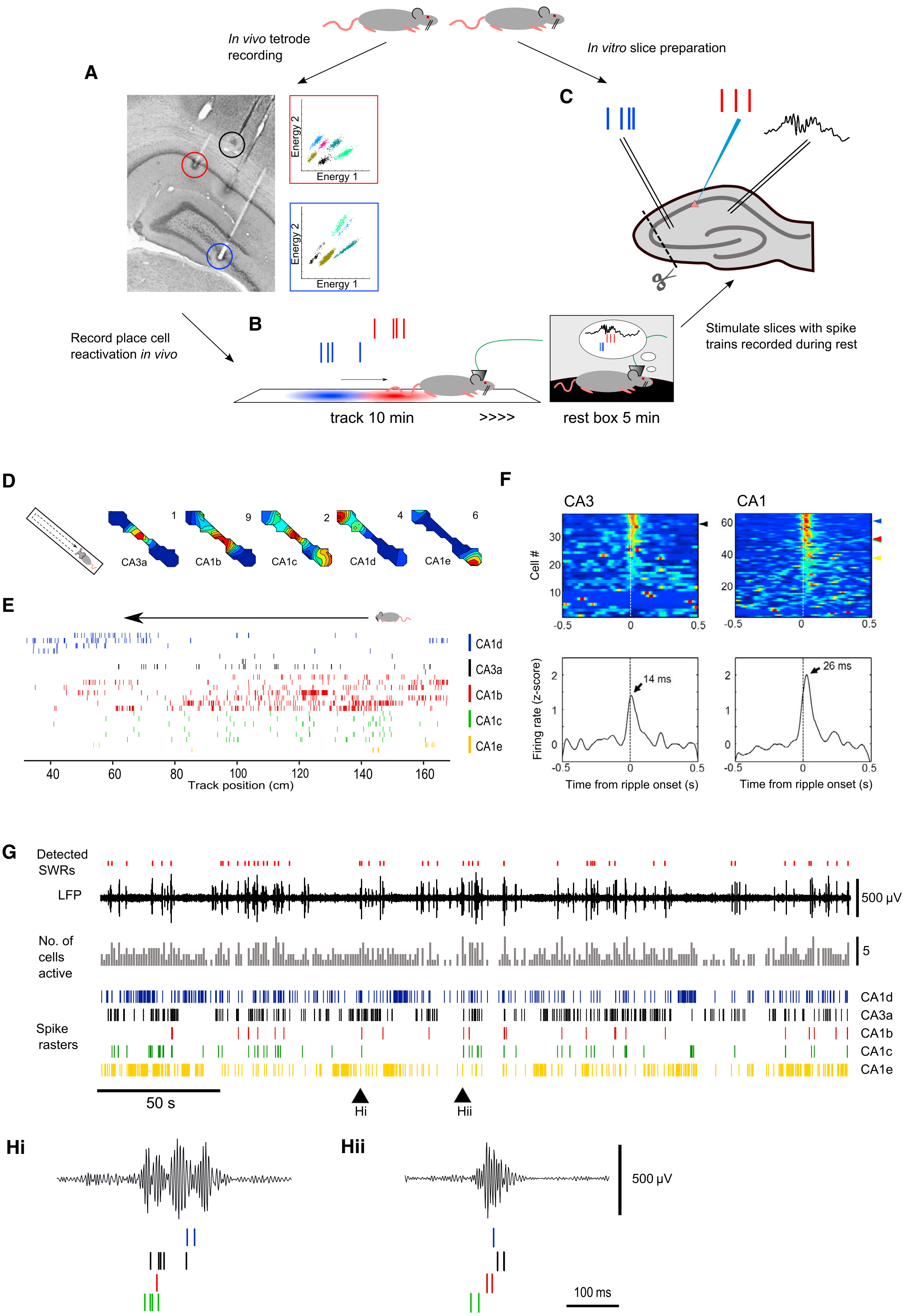

(legend on next page)

of LTP during encoding of memories (Morris et al., 1986; Whitlock et al., 2006).

The reactivation or replay of place cell firing patterns during rest or sleep is associated with transient, high-frequency network oscillations known as sharp-wave ripples (SWRs), which are necessary for normal memory consolidation (Girardeau et al., 2009; Ego-Stengel and Wilson, 2010; Jadhav et al., 2012). Reactivated place cell firing patterns during SWRs undergo time compression by a factor of $\sim$10 when measured across all place cells on a track (Lee and Wilson, 2002), leading to synchronous CA3 and CA1 pyramidal cell firing that is predicted to engage STDP. Therefore, despite reduced levels of cholinergic tone in the hippocampus during rest and sleep, the reactivation of place cell firing patterns during SWRs may support plasticity and memory consolidation in the hippocampus (O'Neill et al., 2010). However, current evidence for LTP induction during memory consolidation falls short of demonstrating that replayed spike patterns induce plasticity; bicuculline-induced bursting in CA3 can induce LTP at SC inputs to CA1 *in vitro* (Buzsáki et al., 1987), and stimulating CA1 pyramidal cells during spontaneous SWR can increase subsequent postsynaptic responsivity *in vivo* (King et al., 1999). Meanwhile, an alternative hypothesis suggests that LTP during sleep could be counterproductive and proposes that synaptic renormalization during sleep may be vital for learning and memory (Grosmark et al., 2012; Tononi and Cirelli, 2014).

Here, we directly test the prediction that reactivated place cell firing patterns induce LTP. We used natural pre- and postsynaptic spike and local field potential (LFP) patterns simultaneously recorded from CA3 and CA1, respectively, during consolidation epochs *in vivo* to control synaptic inputs and postsynaptic spiking in CA1 pyramidal cells recorded *in vitro*. We find that reactivation of place cell firing patterns during SWRs can induce LTP and demonstrate how spike timing in relation to ongoing network activity modulates plasticity.

RESULTS

To address whether synaptic plasticity is induced during reactivation of memory traces, we first sampled CA3 and CA1 place cell firing patterns recorded from adult male Wistar rats *in vivo* during exploration and rest periods (Figure 1A). Unit and LFP activity was recorded while rats explored a familiar linear track for 10 min and were then transferred into a rest box for a 15-min period of quiescence (Figure 1B). Subsequently, a selection of spike trains recorded from CA3 and CA1 place cells during the rest box period on a single day were used to stimulate acute hippocampal slices prepared from naive, non-implanted rats (Figure 1C).

Recording Place Cell Reactivation

To test the plasticity potential of reactivated place cell firing patterns, a subset of five place cells, four from CA1 and one from CA3, recorded in one animal during the first 5 min of the rest box period were selected (Figures 1D–1F). These cells all satisfied criteria identifying them as putative excitatory pyramidal neurons and were selected because they showed clearly defined place fields that were evenly distributed along the length of the track (Figures 1D and 1E). Upon transfer to the rest box, these cells showed typical activity during SWRs, when multiple cells were active within individual SWR time windows (Figure 1F). The median number of spikes per SWR fired by CA3 neurons was 0.22 (first and third quartiles 0.03 and 1.0, respectively); the representative CA3 neuron used for *in vitro* experiments (CA3a in Figure 1) fired an average of 0.45 spikes per SWR. In CA1, neurons fired a median of 0.28 spikes per SWR (first and third quartiles 0.06 and 1.3), with the four exemplars firing averages of 1.42, 0.75, 0.34, and 0.44 spikes per SWR (CA1b–CA1e in Figure 1). The temporal structure of these SWR-associated spiking events commonly reflected the firing sequences observed on the track (Figures 1G and 1H), consistent with reports of remote replay of recent behavioral sequences (Karlsson and Frank, 2009), which is proposed to be important for the consolidation of memory.

Induction of LTP by Reactivation Events

To assess the plasticity potential of these SWR-associated reactivation events, we turned to the *in vitro* hippocampal slice preparation, where the strength of synaptic connections between individual place cells (i.e., CA3 and CA1 pyramidal cells) may be accurately measured. We tested whether the activity of the five place cells recorded during the resting or quiescent phase

Figure 1. Testing the Plasticity Potential of SWR-Associated Reactivation of Behavioral Firing Sequences in CA3 and CA1

(A) Top: adult Wistar rats were used for both *in vivo* and *in vitro* experiments. Hippocampal slices were prepared from naive rats that had not been implanted with tetrodes. Left: example histology shows positions of CA1 (red), CA3 (blue), and local reference (black) tetrodes. Right, top: example clusters recorded on a tetrode located in CA1, example isolation distance and L-ratio for pink cluster was 49.8 and 0.062. Right, bottom: example clusters recorded on a tetrode located in CA3.

(B) Schematic of behavioral paradigm. Rats were allowed to freely explore a familiar track for 10 min, with no reward given, and then transferred into a rest box. LFP and unit activity was recorded throughout.

(C) Spike patterns from CA1 and CA3 cells as well as SWRs detected post hoc were used as the basis for slice stimulation protocols. 400-μm-thick slices were cut from dorsal hippocampus. An incision was made between CA1 and CA3 in each slice.

(D) Firing rate maps of four CA1 and one CA3 place cells while a rat explored a linear track. Warm colors indicate higher firing rates. Mean firing rates shown in top right hand corner of each plot.

(E) Firing position on the track of each cell shown in (B) on inbound runs. Each row represents a single trial. Trials where no spike was detected are not shown.

(F) Peri-stimulus time histograms for all recorded CA3 and CA1 place cells. Cells CA3a and CA1b–CA1e are indicated by color coded arrowheads. Average firing rates for all CA3 and CA1 cells with respect to ripple onset are shown below. On average CA3 cells fired $\sim$12 ms before CA1 cells during SWRs.

(G) Place cell ensemble reactivation took place during SWRs in the rest box. Uppermost trace shows detected SWR time points (red ticks). Black trace shows filtered ripple band LFP (120–240 Hz). Grey trace shows the number of active cells per 1 s bin (maximum of 5). Spike rasters show firing of five place cells during quiet rest.

(H) Two expanded examples of ripple associated reactivation of place cells firing sequences at time points indicated by arrows in (D).

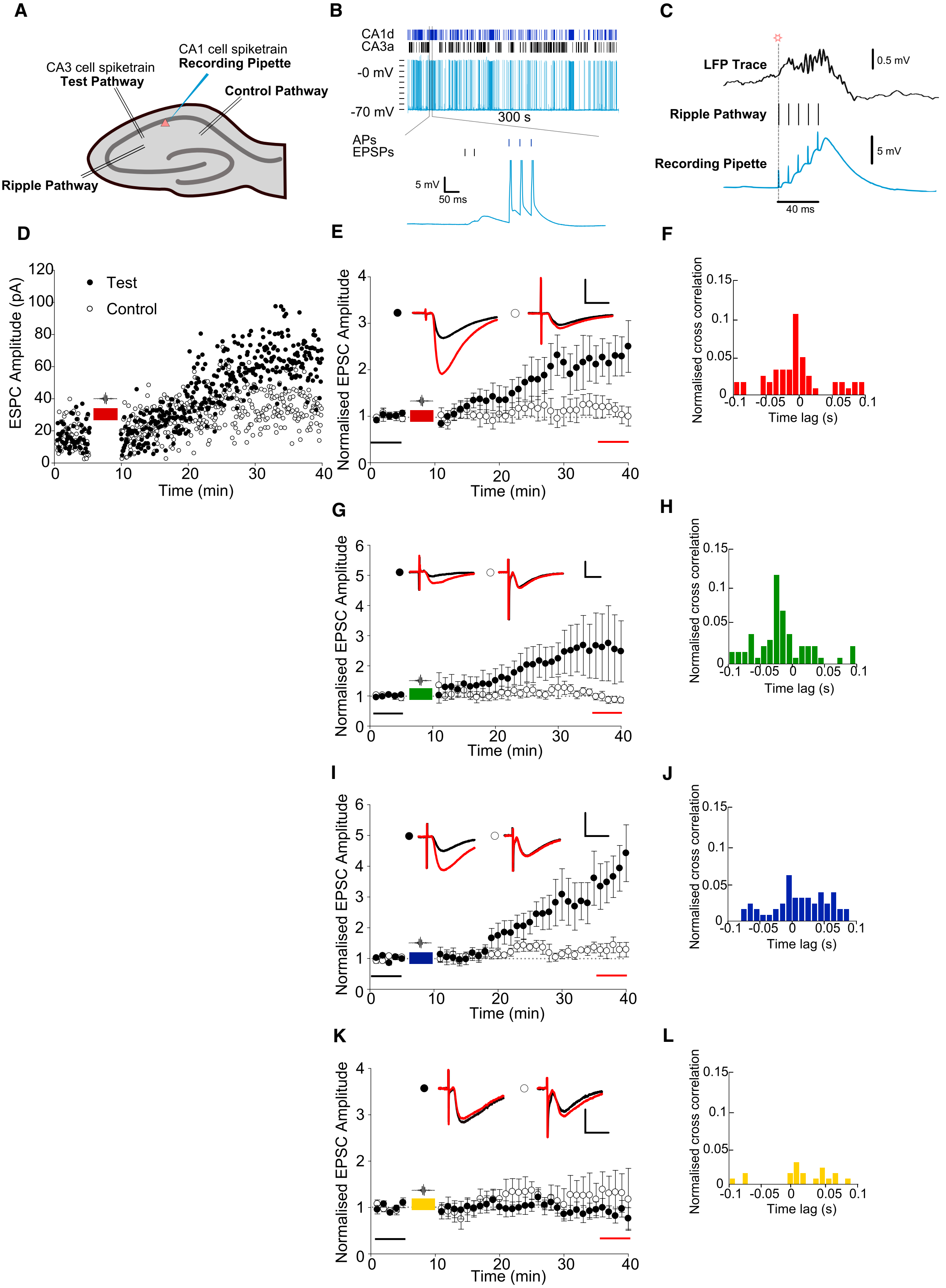

(legend on next page)

(legend on next page)

OPEN
ACCESS
CellPress

were capable of inducing plasticity had they been synaptically coupled (though we made no assumption that these particular place cells were directly interconnected *in vivo*). Given the estimated numbers of place cells active in any one environment and the likely numbers engaged in reactivation (Muller et al., 1987; O'Neill et al., 2008), coupled with the dense connectivity between CA3 and CA1 pyramidal cells (Li et al., 1994), it is not unreasonable to assume that place cells with similar activity profiles to those we have recorded will be synaptically coupled *in vivo* (Isaac et al., 2009).

We made whole-cell patch-clamp recordings from CA1 pyramidal cells in acute hippocampal slices. To replicate the activity seen by synapses *in vivo* during reactivation events, we stimulated these CA1 cells and their SC inputs with patterns of activity recorded from CA1 and CA3 place cells, respectively, during the initial 5 min of the post-run rest period (Figure 2A), as this epoch contained the largest concentration of SWR-associated reactivation events. Timestamps marking when each cell fired during this time period were used to create four induction protocols. In each case, the CA3 spike train provided the presynaptic input and each of the four CA1 cell spike trains provided a different pattern of postsynaptic activity. Synaptic strength at two independent SC-CA1 pathways (control and test) was monitored before and after one of the induction protocols was delivered to the test pathway. Replication of *in vivo* reactivation events was achieved by electrically stimulating a small number of SC axons at CA3 cell spike times to evoke subthreshold excitatory postsynaptic potentials (EPSPs; corresponding to an average baseline excitatory postsynaptic current (EPSC) amplitude of 33.9 ± 5.8 pA for test pathways and 35.1 ± 6.0 pA for control pathways), while action potentials were evoked in the postsynaptic CA1 cell by a brief somatic current injection from the patch pipette at CA1 spike times (Figure 2B). Transient increases in membrane potential caused by phasic excitation that have been observed during SWRs in CA1 pyramidal cells (Maier et al., 2011) were also modeled in the slice preparation; a third independent SC input pathway in stratum radiatum was stimulated with five pulses at 100 Hz at timestamps when SWRs had been detected in the LFP (57 detected in 300 s). The stimulation intensity of this pathway was tuned to match the depolarization envelope duration and amplitude observed *in vivo* (Figure 2C) through synaptic activation of CA1 dendrites (Maier et al., 2011).

Replication of the CA3a-CA1b spike train combination induced test pathway-specific LTP (Figures 2D and 2E; test path, 2.19 ± 0.47; control path, 1.15 ± 0.22; test versus control pathway $p < 0.05$, $n = 8$). These two spike trains were cross-correlated during SWR-associated activity in a 200 ms time window, where peak firing of the CA1b during SWRs occurred 0–10 ms before CA3a (Figure 2F). The CA3a-CA1c combination also induced LTP (Figure 2G; test path, 2.42 ± 0.79; control path, 1.01 ± 0.12; test versus control pathway $p < 0.05$, $n = 8$). Like CA1b, CA1c firing was tightly correlated with CA3a during SWRs within a 200-ms time window, with the CA1 cell most often firing before the CA3 cell (Figure 2H). The largest change in synaptic strength occurred following stimulation with the CA3a-CA1d combination (Figure 2I; test path, 3.36 ± 0.73; control path, 1.28 ± 0.19; test versus control pathway $p < 0.05$, $n = 8$). The cross-correlated firing of CA1d and CA3a during SWRs showed greater numbers of events where the CA3 cell fired before the CA1 cell (Figure 2J). The combination of CA3a-CA1e was the only one not to induce LTP despite having the highest number of spikes occurring during SWRs (Figure 2K; test path, 0.97 ± 0.16; control path, 1.27 ± 0. 43; test versus control pathway $p > 0.05$, $n = 9$). The spiking of CA1e and CA3a was not tightly correlated during SWRs with few CA1 spikes occurring within 30 ms of CA3 spikes (Figure 2L). In all cases, LTP developed slowly, lacking a short-term facilitatory component similar to that previously described for low-frequency STDP in hippocampal slices (Pike et al., 1999; Isaac et al., 2009; Kwag and Paulsen, 2009).

Importance of SWR-Associated Depolarization for Reactivation-Induced LTP

To test the importance of subthreshold depolarizations during SWRs, we repeated these experiments in the absence of

Figure 2. Spike Patterns of CA3 and CA1 Place Cells Taking Part in Remote Ripple-Associated Reactivation during Rest Can Induce LTP in Naive Slices

(A) Schematic of *in vitro* recording setup. A CA1 pyramidal cell was patched and bipolar stimulating electrodes were positioned in the stratum radiatum to provide three independent stimulation pathways (Figure S1). The test pathway simulated the input of a CA3 pyramidal cell to CA1. The ripple pathway was used to simulate transient membrane potential depolarization caused by phasic excitatory input experienced by CA1 pyramidal cells during SWRs.

(B) Method of stimulating slices with CA3 and CA1 cell spike patterns. The induction protocol was recorded in the current clamp configuration. Somatic current injections induced action potentials at CA1 cell time stamps. Electrical stimulation of Schaffer collaterals (SCs) elicited subthreshold EPSPs at CA3 cell timestamps.

(C) SWR-associated synaptic input was achieved using a five-pulse stimulus train (100 Hz) delivered to the ripple pathway at detected SWR onset times.

(D) An example experiment. Test pathway, black circles; control pathway, open circles. Baseline SC stimulation was tuned to elicit subthreshold EPSPs soon after break-in. Baseline EPSCs were recorded every 5 s on the control and test pathway for 5 min in voltage clamp (−70 mV). Spike pattern CA3a-CA1b was delivered between 5 and 10 min in current-clamp configuration. EPSC amplitudes in test and control pathways were recorded for a further 30 min after spike pattern delivery.

(E, G, I, and K) CA3a and CA1b (E), CA3a-CA1c (G), and CA3a-CA1d (I) spike combinations induced pathway-specific LTP, whereas CA3a-CA1e (K) did not. Example EPSC traces from baseline (black) and final 5 min (red) are shown for control and test pathways. Scale bars represent 10 ms and 10 pA (E), 20 pA (G and I), and 30 pA (K).

(F, H, J, and L) CA3a and CA1b (F), CA3a and CA1c (H), CA3a and CA1d (J), and CA3a and CA1e (L) cross-correlation histograms of spike patterns occurring within a time range 50 ms before to 150 ms after the onset of sharp waves plot the time CA1 spikes occurred in a 100 ms time window before and after a CA3 spike (10 ms bins). Cross-correlations are normalized to the total number of CA1 cell spikes occurring within SWRs for each cell: CA1b, 38; CA1c, 45; CA1d, 48; and CA1e, 53.

Data are plotted ± SEM.

OPEN
ACCESS
CellPress

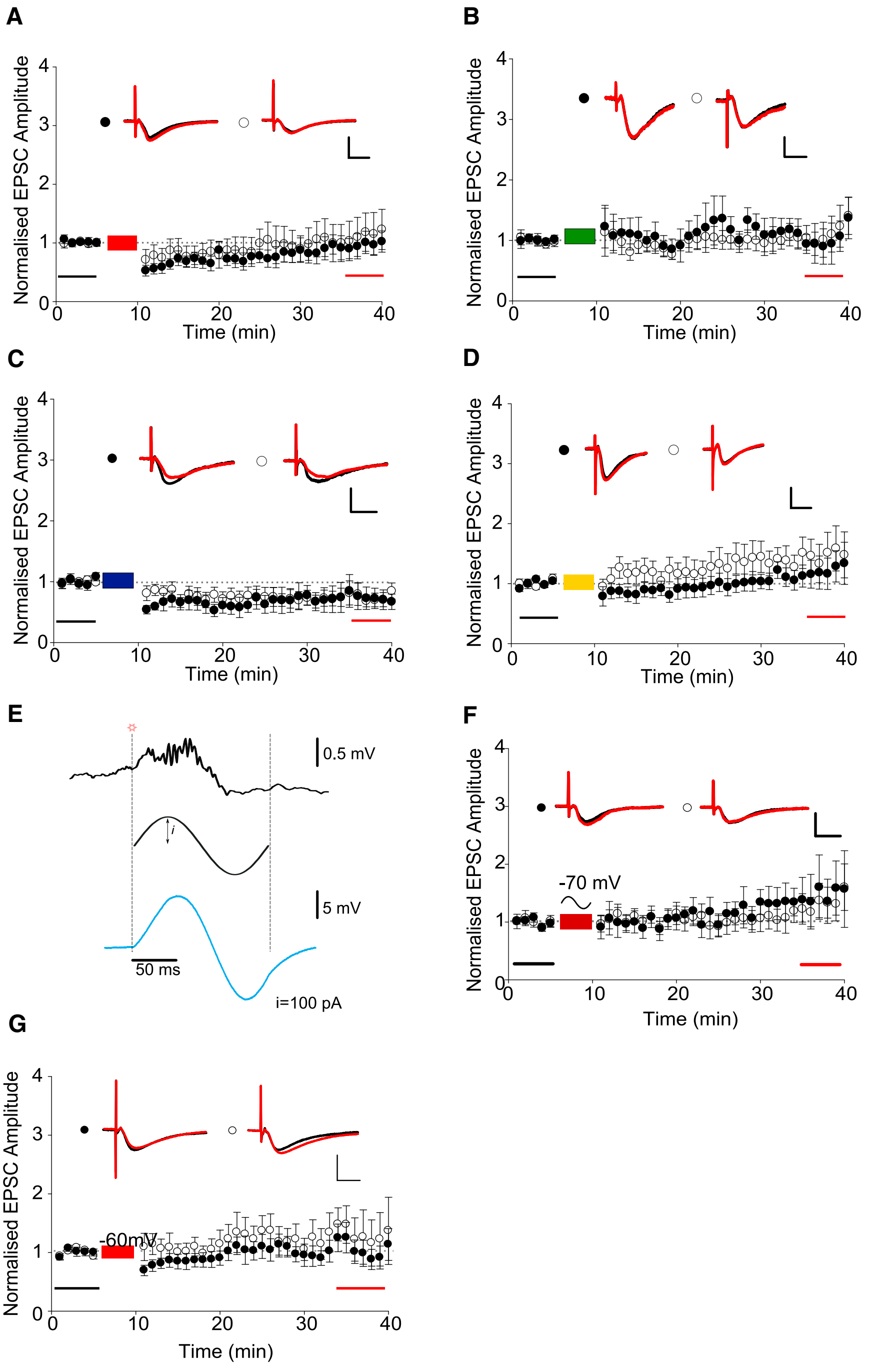

Figure 3. SWR-Associated Synaptic Stimulation Is Required for the Induction of LTP by Reactivated Place Cell Firing Patterns

(A–D) No LTP was induced by CA3a-CA1b (A), CA3a-CA1c (B), CA3a-CA1d (C), or CA3a-CA1e (D) spike combinations in the absence of SWR-associated synaptic stimulation. Example EPSC traces from baseline (black) and final 5 min (red) are shown for control and test pathways.

(E) Modeling the effect of SWR oscillations on cells in CA1 by injecting a sine wave current via the recording pipette at time points at which SWRs were detected in the LFP signal. The frequency of the sine wave was scaled by the duration of the SWR. A maximal current of 100 pA was injected at the peak and valley of the sine wave. Depending on the input resistance of the cell, this gave a maximal membrane potential deflection of between 5 and 10 mV, within the range of that observed *in vivo*.

(F) No LTP was induced by CA3a-CA1b when delivered with sine wave somatic current injections at SWR detection time points. Example EPSC traces from baseline (black) and final 5 min (red) are shown for control and test pathways.

(G) No LTP was induced by CA3a-CA1b when postsynaptic membrane potential was held at −60 mV during the induction protocol. Scale bars represent 10 ms and 20 pA (A), 10 pA (B–D and F), and 50 pA (G).

Data are plotted ± SEM.

SWR-associated depolarization. Spike train stimulation delivered in the absence of SWR-associated synaptic stimulation failed to induce LTP in all cases: CA3a-CA1b (Figure 3A; test path, 0.92 ± 0.19; control path, 1.08 ± 0.32; test versus control pathway p > 0.05, n = 7), CA3a-CA1c (Figure 3B; test path, 1.07 ± 0.25; control path, 1.08 ± 0.25; test versus control pathway p > 0.05, n = 7), CA3a-CA1d (Figure 3C; test path, 0.73 ± 0.19, control path, 0.74 ± 0.13, test versus control pathway p > 0.05, n = 7), and CA3a-CA1e (Figure 3D; test path, 1.13 ± 0.20; control path, 1.43 ± 0.28; test versus control pathway p > 0.05, n = 7).

These data suggest that depolarization associated with SWRs is required to induce LTP using spike patterns recorded during rest. However, it is not clear if depolarization originating from synaptic stimulation is required or whether somatic depolarization is sufficient. To test this, we injected an artificial sine wave current at the soma to replicate the transient membrane potential deflections observed during SWRs *in vivo* (Figure 3E). This method of simulating SWR associated changes in somatic membrane potential failed to facilitate LTP for the CA3a-CA1b spike train combination in the same way as synaptic stimulation (Figure 3F; test path, 1.42 ± 0.40; control path, 1.24 ± 0.27; test versus control pathway p > 0.05, n = 7). Similarly, constant depolarization of the somatic membrane potential to −60 mV during presentation of the CA3a-CA1b spike train failed to facilitate LTP (Figure 3G; test path, 1.03 ± 0.20; control path, 1.29 ± 0.38; test versus control pathway p > 0.05, n = 9). These results indicate that dendritic rather than somatic depolarization during SWRs is the critical factor for LTP induction (Williams and Mitchell, 2008).

OPEN ACCESS
CellPress

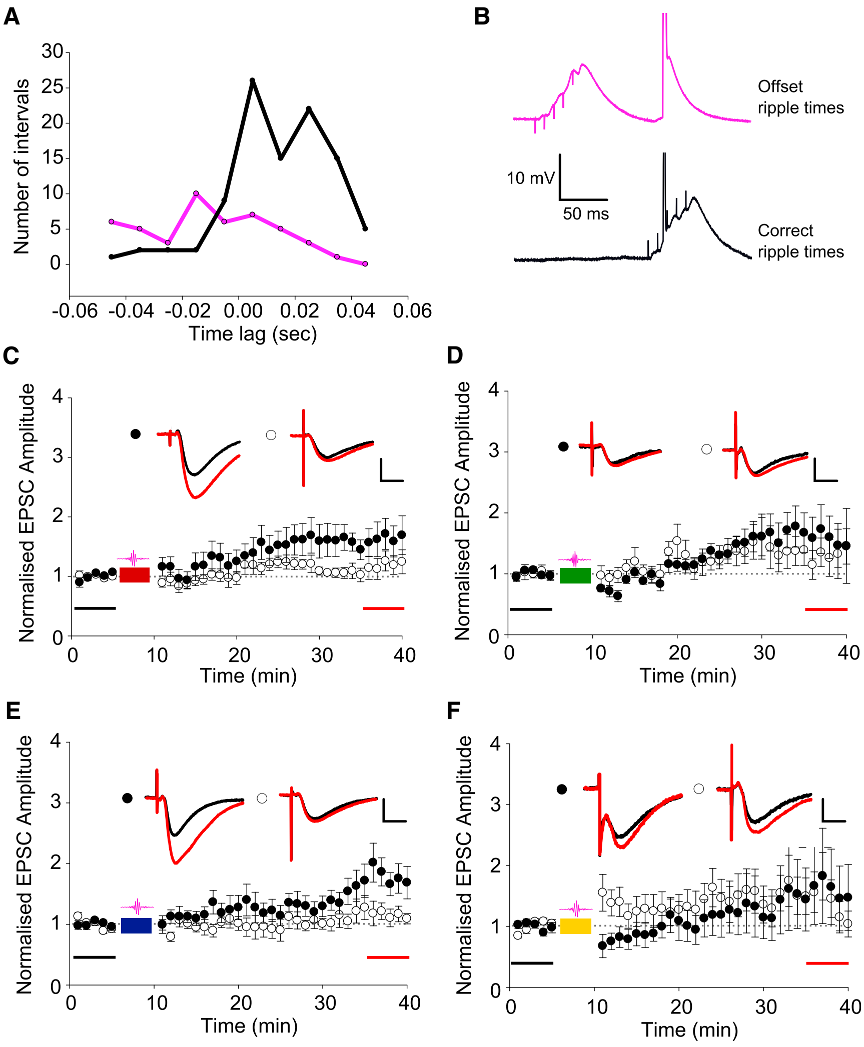

Figure 4. Offsetting Ripple and Spike Times Attenuates or Prevents LTP Induction by Reactivated Place Cell Firing Patterns

(A) CA3 and CA1 spikes (CA1b, c, d, and e combined) occur primarily during SWRs. Co-active CA3-CA1 spiking increases immediately after SWR onset time (black), with no correlation between SWRs and population spiking when SWRs are offset by −100 ms (pink).

(B) Example traces from induction protocol CA3a-CA1d with correct and offset SWR times.

(C–F) LTP induced by CA3a-CA1b (C) or CA3a-CA1d (E) spike combinations was reduced with offset SWR times compared to correct timings (c.f. Figures 2E and 2I). LTP was absent in the case of CA3a-CA1c (D) or CA3a-CA1e (F) spike combinations with offset SWR times. Example EPSC traces from baseline (black) and final 5 min (red) are shown for control and test pathways. Scale bars represent 10 ms and 25 pA (C), 20 pA (D and F), or 30 pA (E).

Data are plotted ± SEM.

Importance of Spike Timing during SWRs for Reactivation-Induced LTP

As well as the location of SWR-associated depolarization, the timing of SWR-associated depolarization is also likely to impact the induction of synaptic plasticity. To test this, we artificially de-coupled the timing of the reactivated spike patterns and the simulated SWR-associated synaptic stimulation.

The *in vivo* data showed that at time points when SWR onsets were detected in the LFP, an increase in the spiking of all five cells used in the spike pattern stimulation experiments was observed (Figure 4A). Offsetting SWRs by shifting them 100 ms earlier relative to the spike times reduced the correlation between spikes and SWRs (King et al., 1999; Ego-Stengel and Wilson, 2010; Jadhav et al., 2012) (Figure 4A). When slices were stimulated with the same spike trains as in Figure 3 but with SWR-associated synaptic stimulation triggered 100 ms early (Figure 4B), LTP was significantly attenuated or not induced at all. Pathway-specific LTP was induced following stimulation with CA3a-CA1b and offset SWRs (Figure 4C; test path, 1.61 ± 0.24; control path, 1.13 ± 0.15; test versus control pathway $p < 0.05$, $n = 9$) but the change in synaptic strength was significantly less than that observed with the correct SWR times (relative change in synaptic strength correct versus offset SWRs $p < 0.05$). Likewise, LTP was induced following stimulation with CA3a-CA1d (Figure 4E; test path, 1.66 ± 0.24; control path, 1.15 ± 0.15; test versus control pathway $p < 0.05$, $n = 8$) but this was also less than that observed with the correct ripple times (relative change in synaptic strength correct versus offset SWRs $p < 0.05$). LTP was not induced following stimulation with CA3a-CA1c (Figure 4D; test path, 1.61 ± 0.34; control path, 1.34 ± 0.32; test versus control pathway $p > 0.05$, $n = 7$) or CA3a-CA1c (Figure 4F; test path, 1.52 ± 0.58; control path, 1.45 ± 0.31; test versus control pathway $p > 0.05$, $n = 7$). The reduction in LTP suggests the timing of spikes within SWRs is critical for LTP induction.

To probe the relationship between spike and SWR timing with higher temporal resolution, we investigated whether the timing of SWR-associated synaptic stimulation could modulate synaptic plasticity induced by artificial spike timing protocols (Figure 5A). In agreement with previous studies (Pike et al., 1999; Buchanan and Mellor, 2007), we found that one subthreshold EPSP followed by one action potential (AP) 10 ms later, repeated 300 times at 5 Hz, did not induce LTP at SC-CA1 synapses (Figure 5B; test path, 1.04 ± 0.12; control path, 1.19 ± 0.21; test versus control pathway $p > 0.05$, $n = 7$). Delivering the same pairing 13 ms after the onset of an SWR-associated synaptic stimulation induced pathway specific LTP (Figure 5C; test path, 2.34 ± 0.43; control path, 1.28 ± 0.16; test versus control pathway $p < 0.05$, $n = 9$). However, delivering the same pairing 40 ms later (53 ms after the onset of the SWR-associated synaptic

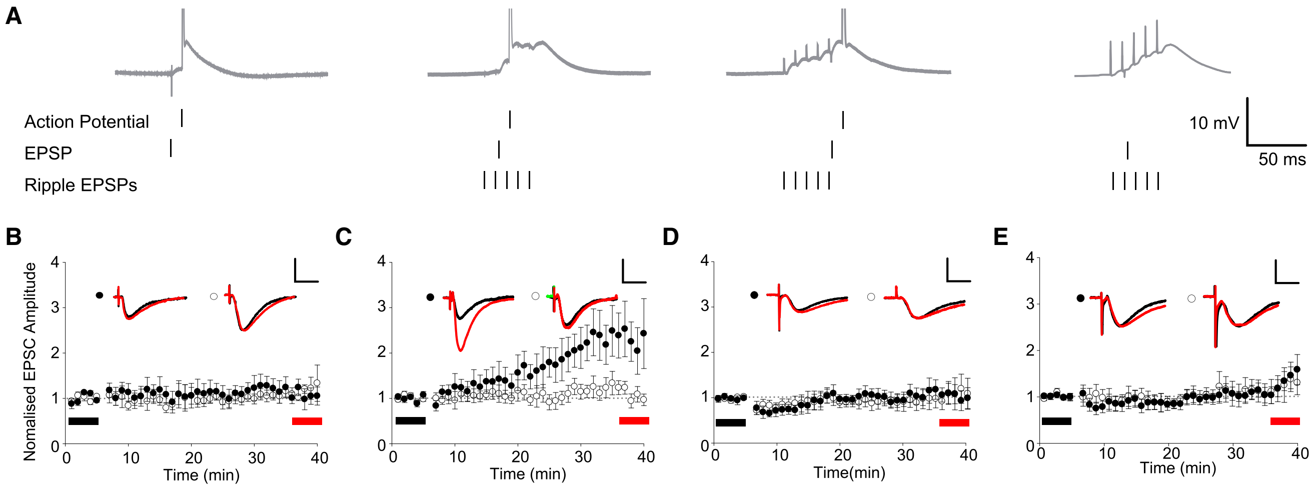

Figure 5. SWR-Associated Synaptic Stimulations Facilitate Spike-Timing Dependent Plasticity Dependent on Infra-ripple Timing

(A) Four artificial induction protocols were tested. Far left: one EPSP followed 10 ms later by one AP (repeated 300 times at 5 Hz). Middle left: the same protocol delivered 13 ms after the onset of a SWR-associated synaptic stimulation. Middle right: the same protocol delivered 53 ms after ripple onset. Right: one EPSP delivered 13 ms after the onset of a SWR-associated synaptic stimulation.

(B) No LTP was induced by one EPSP and one AP.

(C) LTP was induced by one EPSP and one AP delivered near the start of SWR-associated synaptic stimulation.

(D) No LTP was induced by one EPSP and one AP delivered toward the end of the SWR-associated synaptic stimulation.

(E) No LTP was induced by one EPSP delivered near the start of the SWR-associated synaptic stimulation.

Example EPSC traces from baseline (black) and final 5 min (red) are shown for control and test pathways. Scale bars represent 10 ms, 20 pA (B–E). Data are plotted ± SEM.

stimulation) did not result in pathway-specific LTP (Figure 5D; test path, 1.37 ± 0.33; control path, 1.04 ± 0.36; test versus control pathway $p > 0.05$, $n = 6$). This is not simply a form of associative plasticity coupling the strong ripple pathway with the weak test pathway, as one EPSP alone delivered to the test pathway 13 ms after SWR onset failed to induce LTP (Figure 5E; test path, 1.34 ± 0.28; control path, 1.18 ± 0.26; test versus control pathway $p > 0.05$, $n = 6$). Together, these data show that the timing of coincident pre- and postsynaptic activity in relation to the SWR-associated synaptic stimulation is critical for LTP induction. Furthermore, it suggests that CA3-CA1 spike pairs in the first portion of an SWR are the most important for inducing LTP.

Properties of Plasticity-Inducing Spike Trains

We found that the spike train capable of inducing the largest change in synaptic strength (CA3a-CA1d) contained eight such plasticity-potent events. These events were classified as a CA3 spike followed less than 30 ms later by a CA1 spike or burst and occurred either just before (less than 30% of the SWR's duration before onset time) or during the first part of the SWR duration (in the first 60% of a SWR) (Figure 6A). To test whether these events were necessary for LTP induction, we removed the ten CA1 spikes that constituted these events from spike train CA3a-CA1d (Figure 6B). No LTP was induced by this spike train following the removal of the ten CA1 spikes (Figure 6C; test path, 1.08 ± 0.17; control path, 0.99 ± 0.25; test versus control pathway $p > 0.05$, $n = 6$). Next, we tested whether these spike events were sufficient for LTP induction by delivering these events alone. Again, no LTP was induced (Figure 6D; test path, 1.51 ± 0.26; control path, 1.28 ± 0.26; test versus control pathway $p > 0.05$, $n = 6$). Nor was any LTP induced when the intact spike train, which had previously induced robust LTP (Figure 2I), was used in the presence of the NMDA receptor antagonist DL-AP5 (Figure 6E; test path, 0.97 ± 0.20; control path, 0.87 ± 0.19; test versus control pathway $p > 0.05$, $n = 6$). Hence, based on this example, spiking events such as those defined in Figure 6A are necessary, but not sufficient, for the induction of NMDA-receptor-dependent LTP.

To test if the results from this example pair of place cells might be generalized, we analyzed the number of such necessary spike pairings within SWRs in each spike train protocol. The number of necessary spike pairings within SWRs showed a strong correlation with the change in synaptic strength induced by these spike trains (Figure 7A; $r^2 = 0.89$), supporting a model where LTP-competent pairings have a probability of inducing stepwise changes in synaptic strength (O'Connor et al., 2005). Other factors that might also predict change in synaptic strength, such as CA1 bursts following CA3 spikes or total number of CA1 spikes, did not correlate with induced change in synaptic strength (Figures 7B and 7C). These results support the conclusion that pairs of CA3 and CA1 spikes that occur within a short time window around the start of SWRs are the predominant factor influencing LTP induction.

DISCUSSION

Place cell firing sequences are reactivated at compressed timescales during hippocampal SWRs (Nádasdy et al., 1999; Lee and Wilson, 2002; Foster and Wilson, 2006; Diba and Buzsáki, 2007;

OPEN ACCESS
CellPress

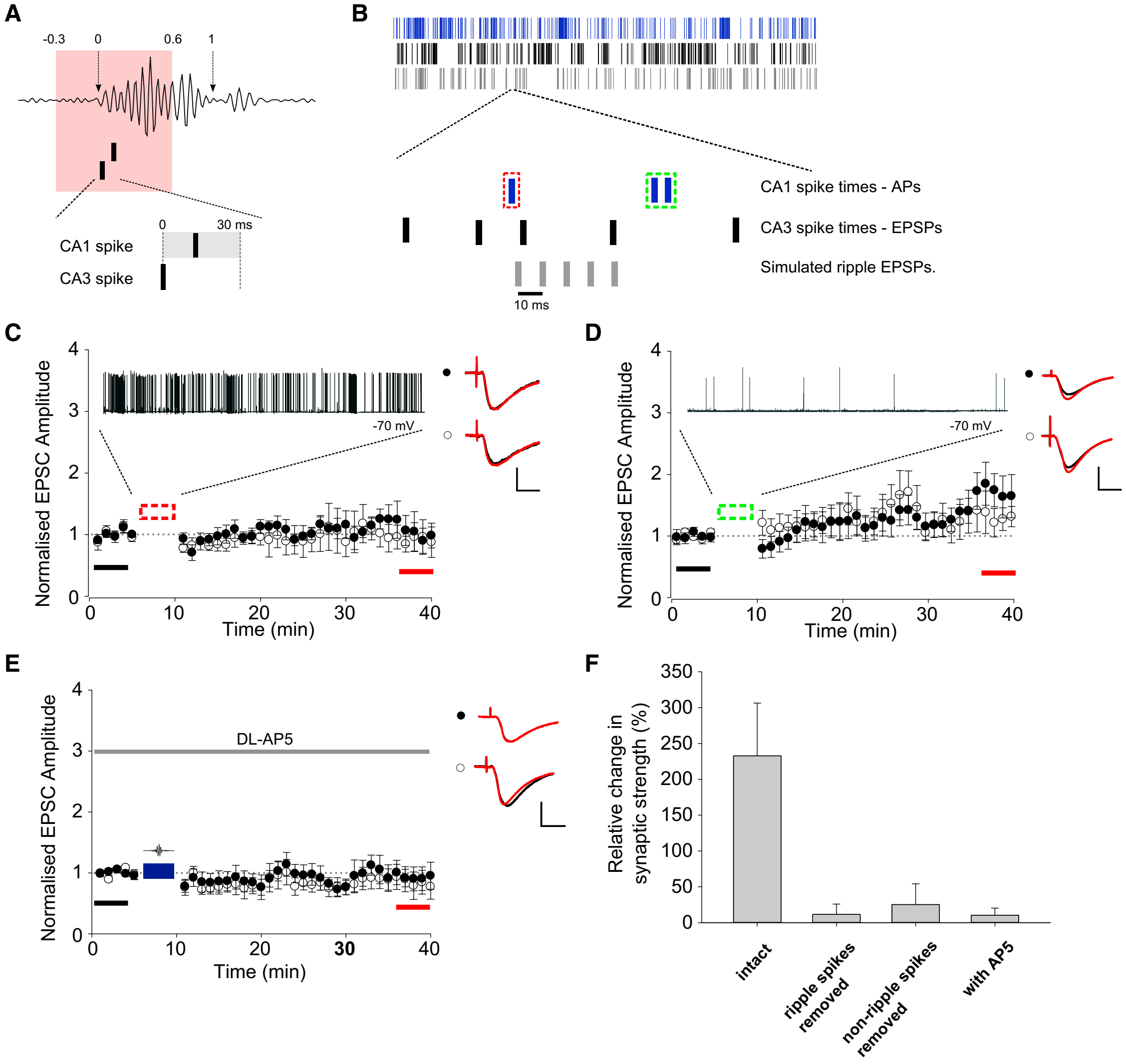

Figure 6. Causal CA3-CA1 Spiking Events during SWRs Are Necessary, but Not Sufficient, to Induce LTP

(A) Casual events where a CA3 spike was followed by a CA1 spike/burst <30 ms later and occurred in the time window shown by red rectangle were identified. The time window was defined as being from 30% of the total SWR duration before onset and 60% of the total SWR duration after onset.

(B) Top: rasterplot of spike train CA3a-CA1d. Below: expanded section of the spike train that includes a predicted plasticity-inducing event as defined in (A). Timestamps highlighted by dashed red line were removed in experiment shown in (C). Timestamps highlighted by dashed green line were removed in experiment shown in (D).

(C) No LTP was induced by CA3a-CA1d spike combination when ten spikes occurring during plasticity predictive events were removed from the CA1d spike train. Trace above plot shows induction protocol recorded in current clamp.

(D) No LTP was induced by CA3a-CA1d induction protocol when all but the identified plasticity-predictive events were removed from the CA3 and CA1 spike train. Trace above plot shows induction protocol recorded in current clamp.

(E) No LTP was induced by CA3a-CA1d induction protocol (as shown in Figure 3I) in the presence of DL-AP5 (100 μM).

(F) Bar graph summarizes data shown in Figure 3I and (C)–(E).

Example EPSC traces from baseline (black) and final 5 min (red) are shown for control and test pathways. Scale bars represent 10 ms, 10 pA. Data are plotted ± SEM.

Davidson et al., 2009; Karlsson and Frank, 2009), generating conditions compatible with induction of STDP (Bi and Poo, 1998; Debanne et al., 1998; Wittenberg and Wang, 2006; Buchanan and Mellor, 2007), and thus facilitating learning and memory (Girardeau et al., 2009; Ego-Stengel and Wilson, 2010; Jadhav et al., 2012). However, direct demonstration of synaptic plasticity induced by replayed activity during SWRs has not previously been provided. In this study, we have formally tested these important hypotheses and found that reactivated place cell firing patterns are able to induce LTP at SC-CA1 synapses but require the additional excitatory synaptic input that CA1 cells receive during SWRs *in vivo*. Causal spike pairs occurring near SWR onset times are necessary for the induction of plasticity, indicating that infra-ripple spike timing may be a critical

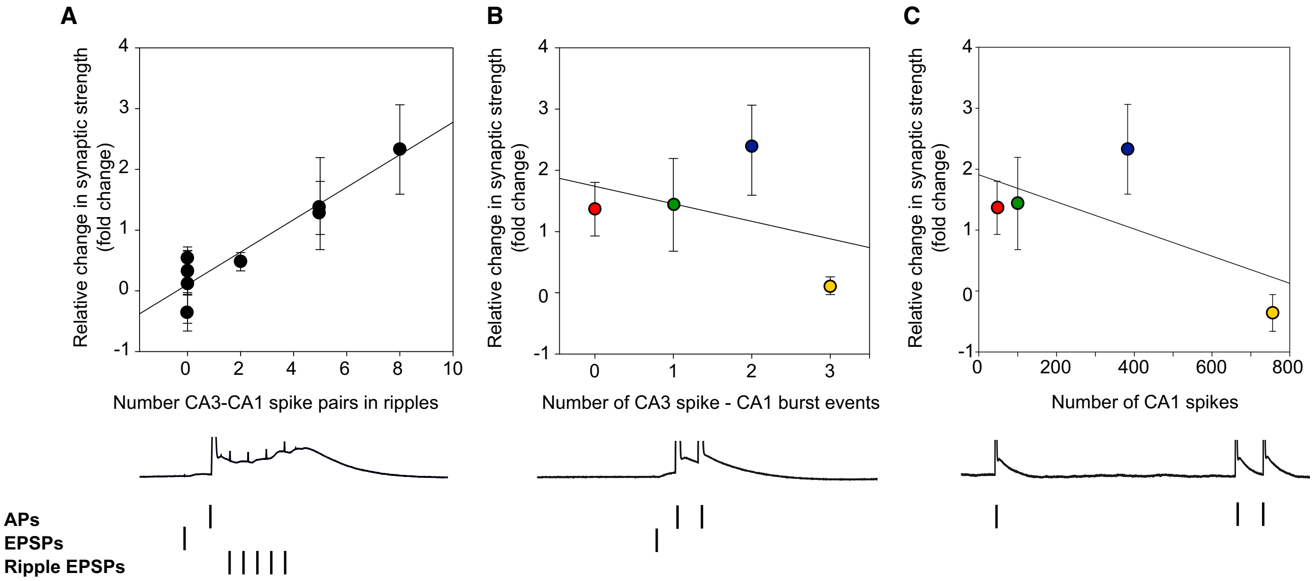

Figure 7. The Number of Coincident CA3-CA1 Causal Spiking Events during SWRs Is Highly Predictive of the Plasticity-Inducing Potential of a Spike Pattern

(A) A strong correlation between relative change in synaptic strength induced by all spike combinations and the number of causal CA3-CA1 spike pairs during SWRs in each protocol, as defined in Figure 6A (r^2 = 0.89).

(B) No correlation between the relative change in synaptic strength induced by the spike combinations used in the experiments shown in Figure 2 and the number of CA3 spikes followed <30 ms later by CA1 cell bursts in each spike combination.

(C) No correlation between the relative change in synaptic strength induced by the spike combinations used in the experiments shown in Figure 2 and the total number of CA1 spikes in each spike train.

determinant of plasticity *in vivo*. We hypothesize that this form of synaptic plasticity has an important function in consolidating and maintaining hippocampal representations of space.

Of the representative spike train pairs tested here, which were simultaneously recorded from CA3 and CA1 place cells during a post-run rest period, three were capable of inducing LTP given SWR-associated synaptic stimulation. Though all the tested spike trains had tightly cross-correlated spiking, CA3a-CA1e did not have any causal events near SWR onset and did not induce LTP under any conditions, supporting the conclusion that the timing of spikes within SWRs is critical for LTP induction. This might be expected given that CA3a and CA1e had the most distant place fields for any of the CA3-CA1 pairs and therefore their reactivation is expected to span the duration of SWRs. Interestingly, even though there were plenty of acausal CA3 and CA1 spike timings, none of the tested spike trains induced pathway specific LTD. This is similar to the situation for plasticity induced by place cell firing patterns during exploration (Isaac et al., 2009) and might be explained by a dominance for LTP over LTD or the lack of STD-LTD exhibited at mature SC-CA1 synapses (Buchanan and Mellor, 2007; Tigaret et al., 2016) compared to immature synapses which exhibit presynaptically expressed STD-LTD (Sjöström et al., 2003; Bender et al., 2006; Nevian and Sakmann, 2006; Rodríguez-Moreno and Paulsen, 2008; Min and Nevian, 2012).

Artificially shifting the timing of SWR-associated synaptic stimulation reduced or abolished LTP, indicating an important, time-sensitive interaction between structured place cell firing patterns and SWR-associated synaptic input. Indeed, we found that SWR-associated synaptic stimulation can powerfully modulate STDP at mature SC-CA1 synapses. These findings demonstrate how the temporal structure of reactivated place cell firing patterns interacts dynamically with network oscillations to sculpt plasticity in the hippocampus, providing important data to inform models of the impact of plasticity's impact on place cell firing patterns (Mehta et al., 2000; Bush et al., 2010b). Indeed, previous models have demonstrated the importance of bursts of coincident dendritic activity to induce LTP at distal synapses in the absence of strong back-propagating action potentials (Kumar and Mehta, 2011). Modeling studies also highlight the importance of spike timing to generate sufficient NMDAR activation and subsequent Ca^{2+} influx into dendritic spines, which strengthens synaptic connectivity between place cells with overlapping place fields and creates place cell assemblies (Mehta et al., 2000; Bush et al., 2010b). However, such models rely on experimental data to constrain the underlying STDP rules using either phenomenological (Clopath et al., 2010) or biophysical Ca^{2+}-based models (Shouval et al., 2002; Rackham et al., 2010; Kumar and Mehta, 2011). The latter make assumptions about the relationship between total spine Ca^{2+} and LTP/LTD based on the Ca^{2+} control hypothesis, which has been challenged by recent experimental evidence suggesting that the relative timing of Ca^{2+} release from distinct Ca^{2+} sources within dendritic spines, including NMDA receptors and voltage-gated Ca^{2+} channels, is key to the induction of STDP (Nevian and Sakmann, 2006; Tigaret et al., 2016). In many modeling studies, further

assumptions are made for the existence of STD-LTD that are critical for the stability of the model output but as discussed above may be incorrect and therefore require reappraisal. Thus, our data represent important information to update the assumptions underlying STDP modeling for mature SC-CA1 synapses that may reveal new insights into the role of synaptic plasticity in place cell assembly formation.

How does SWR-associated excitatory input at one synaptic locus influence the plasticity inducing potential of pre- and postsynaptic activity patterns at a synaptic connection between two place cells? One explanation is that the coincident activation of two independent synaptic inputs induces an associative form of LTP. However, this is unlikely to be the case, since the stimulation of the test and ripple synaptic inputs in the absence of postsynaptic action potentials was insufficient to induce LTP (Figure 5E). Alternatively, the additional synaptic input and resulting dendritic depolarization may increase the amplitude of backpropagating action potentials facilitating the activation of NMDA receptors and LTP induction (Magee and Johnston, 1997). One implication is that SWR-associated excitatory synaptic input enhances dendritic excitability and therefore lowers the threshold for induction of plasticity. This is supported by many studies showing that dendritic depolarization facilitates LTP induced by spike pairings (reviewed in Williams et al., 2007) and that dendritic depolarization and LTP may be enhanced by the frequency of spike pairings (Sjöström et al., 2001; Carlisle et al., 2008). Furthermore, dendritic depolarization and LTP may be enhanced by coincident synaptic inputs from SC and temperoammonic pathways leading in some cases to dendritic plateau potentials, which are strong predictors of synaptic plasticity (Golding et al., 2002). Plateau potentials occurring during exploration have been shown to be important for shaping place cell activity *in vivo*, presumably via the induction of synaptic plasticity (Gambino et al., 2014; Bittner et al., 2015; Sheffield and Dombeck, 2015). While these plateau potentials generated in distal dendrites by temperoammonic and SC input to CA1 pyramidal neurons are critical for some forms of plasticity induced during awake behavior, we did not see plateau potentials in our recordings, and during SWRs, plateau potentials are largely absent (Bittner et al., 2015). We conclude that an enhancement of dendritic depolarization facilitates LTP induction during SWRs but is not reliant on the generation of plateau potentials.

In the context of dendritic depolarization, the contribution of inhibitory synaptic inputs associated with SWRs is also highly relevant as a mechanism of potentially counteracting depolarization and inhibiting LTP induction (Groen et al., 2014). Inhibitory inputs during SWRs are principally located on somatic rather than dendritic compartments (Klausberger et al., 2003; Varga et al., 2012), and our results suggest that reducing somatic excitability does not significantly alter the threshold for plasticity induced during SWRs. Furthermore, it has been shown that stimulation of CA1 pyramidal neurons during SWRs *in vivo* enhances subsequent CA1 excitability, suggesting that synaptic plasticity during SWRs may be induced in the presence of inhibition (Buzsáki et al., 1987; King et al., 1999). However, the role of precisely targeted inhibitory input during SWRs in regulating synaptic plasticity remains to be elucidated. The enhancement of dendritic excitability during SWRs superficially predicts that late causal spiking in SWRs will be more likely to induce plasticity. One possible explanation for the importance of early rather than late causal spiking is the slow onset of voltage- and Ca^{2+}-dependent potassium conductances that may reduce dendritic excitability toward the end of SWRs. An example is Ca^{2+}-dependent potassium conductances (SK channels) that are present in dendritic spines where they closely regulate NMDA receptor activity (Faber et al., 2005; Ngo-Anh et al., 2005; Bloodgood and Sabatini, 2007; Buchanan et al., 2010).

It has been suggested that ripple-associated replay in the hippocampus allows recently encoded spatial engrams to become consolidated though synaptic plasticity (O'Neill et al., 2010; Carr et al., 2011; Sadowski et al., 2011). Neurons representing multiple elements of the engram will fire together and therefore "wire together." However, since ripples boost firing rates across much of the CA3 and CA1 pyramidal cell network, ensuring plasticity only occurs at specific synapses may be problematic. The intra-ripple timing dependent plasticity we demonstrate in this study addresses this issue. Recently active cell assemblies undergo a degree of potentiation during behavior (Isaac et al., 2009), with enhanced connection strengths subsequently influencing replay activity during rest and sleep. In addition, the enhanced connectivity will make cells within the recently active assembly more excitable, hence more likely to fire immediately after ripple onset. Non-participating cells or cells that have distant place fields and are therefore not tightly bound into the ensemble during exploration (e.g., CA1e) may tend to fire later in the ripple oscillation and not undergo plasticity. In this way, ripples can act to promote and tune synaptic plasticity within the hippocampal network, enhancing the signal-to-noise ratio within the neural code. Previous studies have reported both forward and reverse replay during rest (Foster and Wilson, 2006; Diba and Buzsáki, 2007). Where extended replay sequences are concerned, our data predict that reverse replay would enhance the connectivity of place cells encoding proximal locations whereas forward replay would enhance connectivity between cells encoding the beginning of a trajectory. The balance of forward and reverse replay could therefore reflect task demands, with forward replay occurring after an animal leaves a reward location and reverse replay more likely when they arrive at a new one.

Sleep has an important role in learning and memory, but the precise nature of this role is a matter of debate. Cuing the reactivation of recently acquired information during slow-wave sleep can enhance memory (Gais et al., 2006; Rudoy et al., 2009), suggesting that replay in the hippocampus may support memory consolidation (Born et al., 2006; Marshall and Born, 2007). Others suggest that sleep provides a vital opportunity for synaptic downscaling (Vyazovskiy et al., 2008; Maret et al., 2011) following cumulative potentiation during wakefulness and that further potentiation during sleep could harm memory encoding (Tononi and Cirelli, 2014). Our data suggest that brain activity during quiescence, a state somewhere between sleep and wakefulness, may enable the connectivity of specific spatial engrams to be enhanced prior to sleep, evidence that is compatible with both theories of sleep function. These engrams may be preferentially reactivated and consolidated in the cortex during sleep (Rosanova and Ulrich, 2005; Chauvette et al., 2012); if synaptic downscaling occurs, signal-to-noise ratio will be improved,

and these representations will become more salient (Grosmark et al., 2012).

In conclusion, our results show that reactivated place cell firing patterns can induce LTP when accompanied by SWR-associated synaptic input. These data confirm a widely held assumption that reactivation during SWRs promotes synaptic plasticity. They also suggest an active role for phasic excitatory input during SWRs in tuning STDP *in vivo*. In future studies, it will be important to investigate how SWR-dependent STDP can influence learning and memory directly.

EXPERIMENTAL PROCEDURES

Tetrode Implantation

All procedures were conducted in accordance with the UK Animals (Scientific Procedures) Act, 1986 and with the approval of the University of Bristol ethics committee. Three adult (350–450 g) male Wistar rats (Charles River) were chronically implanted with 19 extracellular tetrode recording electrodes (8 into CA3, 8 into CA1, and 3 into the white matter of the fimbria fornix in the right dorsal hippocampus [−3.6 mm, +2.2 mm from bregma]) under isoflurane recovery anesthesia. During the 7–21 days following surgery, the independently moveable tetrodes were lowered into the brain, targeting the pyramidal cell layer in the dorsal CA1 and CA3 (verified by the characteristic burst mode of single-unit firing and the presence of large-amplitude SWR events in the LFP signal). Recordings were made using a Digital Lynx system (Neuralynx). LFPs (sampled at 2 kHz and filtered between 0.1–475 Hz) and extracellular action potentials (sampled at 30 kHz and filtered between 0.6–6 kHz) were recorded differentially using local references in the white matter overlying the hippocampus. All channels were grounded to two screws placed in the skull overlying the cerebellum. Final tetrode tip positions were verified histologically by identifying sites of electrolytic lesions (Figure 1C) made at the end of experimental procedures under terminal sodium pentobarbital anesthesia.

Recording Protocols

Animals were trained to run back and forth on a 200 × 10 cm linear track for a small food reward for a period of 14 days prior to surgery. During these 14 days, animals were food restricted to 90% of free feeding body weight. Recording sessions began once electrodes were in position 21 days after surgery. In a familiar recording room, animals were first placed on a raised platform in a rest box for a 15 min period before being moved to the track where they were allowed to explore freely for 10 min. Animals were then placed back in the sleep box for a further 15 min period. Animals did not receive food reward on the track and were not food restricted prior to recording sessions. Animal movement and behavior was monitored continuously by video. Position on the track was tracked using light-emitting diodes attached to a powered headstage (Cheetah Software; Neuralynx).

In Vivo Data Analysis

All data were processed in MatLab (MATHWORKS) unless stated otherwise. Single units were isolated manually offline using MClust 3.5 (A.D. Redish, available at http://redishlab.neuroscience.umn.edu/MClust/MClust.html); inclusion criteria were set to isolation distance > 15.0 and L-ratio < 0.35. Putative pyramidal cells were classified on the basis of the spike width, waveform, and mean firing rate. Ripples were detected offline in the LFP recorded on one CA1 channel. Raw LFP signal was filtered between 120 and 250 Hz, and deflections in the ripple power envelope greater than 5 SDs from the mean were classified as ripple events. Ripple start times were defined locally as when ripple power exceeded 2 SDs. Samples of raw LFP and detected ripple times were compared manually to verify detection fidelity. For place cell analysis, the track area was divided into 10 × 10 cm bins, and mean firing rates for each neuron in each bin were calculated.

Slice Preparation

Brain slices were prepared from adult (10- to 12-week-old) male Wistar rats following a lethal dose of anesthetic (isoflurane inhalation). Brains were dissected in ice-cold cutting solution containing 119 mM NaCl, 2.5 mM KCl, 1 mM NaH_2PO_4, 26.2 mM $NaHCO_3$, 10 mM glucose, 1.3 mM $CaCl_2$, and 2.5 mM $MgSO_4$ equilibrated with 95% O_2 and 5% CO_2. Coronal slices 300–400 μm thick were cut from the dorsal hippocampus using a vibratome (Leica LS1200), and slices were incubated in artificial cerebrospinal fluid (aCSF) containing 119 mM NaCl, 2.5 mM KCl, 1 mM NaH_2PO_4, 26.2 mM $NaHCO_3$, 10 mM glucose, 2.5 mM $CaCl_2$, and 1.3 mM $MgSO_4$ at 36°C for 30 min and then stored at room temperature until use. Before being transferred to the recording chamber, a cut was made between CA3 and CA1.

Whole-Cell Patch-Clamp Recordings

Recordings were made in a submerged chamber perfused with aCSF (as above) at 34°C with the addition of 50 μM picrotoxin to block $GABA_A$-receptor-mediated transmission to enable accurate measurement of monosynaptic excitatory connections between hippocampal pyramidal cells. CA1 pyramidal cells were visualized using infrared DIC optics on an Olympus BX-51 microscope. Patch electrodes with a resistance of 4–5 MΩ were pulled from borosilicate filamented glass capillaries (Harvard Apparatus) using a vertical puller (PC-10, Narashige). Pipettes were filled with intracellular solution containing 120 mM $KMeSO_3$, 10 mM HEPES, 0.2 mM EGTA, 4 mM Mg-ATP, 0.3 mM Na-GTP, 8 mM NaCl, and 10 mM KCl (pH 7.4), 280–285 mOsm.

Recordings from CA1 pyramidal neurons were made with an Axopatch 200B or a Multiclamp 700A amplifier (Molecular Devices, USA), filtered at 4–5 kHz and digitized at 10 kHz using a data acquisition board and signal acquisition software (CED). Cells were voltage clamped at −70 mV (junction potential correction of −11 mV not accounted for). Series resistance was monitored throughout the experiments and cells that showed a >20% change were discarded.

Synaptic responses were evoked in control and test pathways with 100-μs-square voltage steps applied at 0.1 Hz through two bipolar stimulating electrodes located in stratum radiatum. A third stimulation pathway in stratum radiatum was used to simulate SWR-associated synaptic stimulation and dendritic depolarization during plasticity induction. The three pathways were tested regularly to ensure independence by paired-pulse protocols (Figure S1). Postsynaptic action potentials were initiated through somatic current injections (2 ms duration, 2 nA amplitude).

Replay of Place Cell Spike Patterns

Small amplitude EPSCs (typically 20–40 pA) were recorded in visually identified CA1 pyramidal cells voltage clamped at −70 mV. The stimulation intensity of each input pathway was tuned to elicit sub-threshold EPSPs following a five pulses at 100 Hz stimulus prior to baseline recording. EPSCs were recorded in voltage clamp from two independent pathways for a baseline period of 5 min. Spike train stimulation and spike timing protocols were applied after the neurons were switched to current-clamp mode within 10 min of reaching the whole-cell configuration. The resting membrane potential of the neurons was −70.0 ± 0.5 mV. Following induction, responses to both test and control pathway stimulation were monitored for a further 30–34 min in voltage-clamp mode (Figure 2D).

In Vitro Data Analysis

Measurements were made from averages of six traces to give one data point per minute. Average data are presented as mean ± SEM. Data were normalized to the average baseline response. Data comparisons were made between test and control pathways at 25–30 min after plasticity induction using Student's paired two-tailed t test with a significance level of $p < 0.05$. For between-dataset comparisons of plasticity induction, relative change in synaptic strength (mean test minus mean control pathway response during the final 5 min of the experiment) was calculated for each experiment and values compared using an unpaired Student's two-tailed t test.

SUPPLEMENTAL INFORMATION

Supplemental Information includes one figure and can be found with this article online at http://dx.doi.org/10.1016/j.celrep.2016.01.061.

AUTHOR CONTRIBUTIONS

J.H.L.P.S. conducted the experiments. J.H.L.P.S., M.W.J., and J.R.M. designed and analyzed experiments and wrote the paper.

ACKNOWLEDGMENTS

We thank A. Frick, U. Bartsch and members of the M.W.J. and J.R.M. labs for helpful discussion and R. Bogacz and Z. Bashir for comments on previous versions of the manuscript. This work was funded by the Medical Research Council, UK.

Received: December 10, 2014
Revised: October 16, 2015
Accepted: January 19, 2016
Published: February 18, 2016

REFERENCES

Bender, V.A., Bender, K.J., Brasier, D.J., and Feldman, D.E. (2006). Two coincidence detectors for spike timing-dependent plasticity in somatosensory cortex. J. Neurosci. *26*, 4166–4177.

Bi, G.Q., and Poo, M.M. (1998). Synaptic modifications in cultured hippocampal neurons: dependence on spike timing, synaptic strength, and postsynaptic cell type. J. Neurosci. *18*, 10464–10472.

Bittner, K.C., Grienberger, C., Vaidya, S.P., Milstein, A.D., Macklin, J.J., Suh, J., Tonegawa, S., and Magee, J.C. (2015). Conjunctive input processing drives feature selectivity in hippocampal CA1 neurons. Nat. Neurosci. *18*, 1133–1142.

Bliss, T.V., and Collingridge, G.L. (1993). A synaptic model of memory: long-term potentiation in the hippocampus. Nature *361*, 31–39.

Bloodgood, B.L., and Sabatini, B.L. (2007). Nonlinear regulation of unitary synaptic signals by CaV(2.3) voltage-sensitive calcium channels located in dendritic spines. Neuron *53*, 249–260.

Born, J., Rasch, B., and Gais, S. (2006). Sleep to remember. Neuroscientist *12*, 410–424.

Buchanan, K.A., and Mellor, J.R. (2007). The development of synaptic plasticity induction rules and the requirement for postsynaptic spikes in rat hippocampal CA1 pyramidal neurones. J. Physiol. *585*, 429–445.

Buchanan, K.A., and Mellor, J.R. (2010). The activity requirements for spike timing-dependent plasticity in the hippocampus. Front. Synaptic Neurosci. *2*, 11.

Buchanan, K.A., Petrovic, M.M., Chamberlain, S.E., Marrion, N.V., and Mellor, J.R. (2010). Facilitation of long-term potentiation by muscarinic M(1) receptors is mediated by inhibition of SK channels. Neuron *68*, 948–963.

Bush, D., Philippides, A., Husbands, P., and O'Shea, M. (2010a). Dual coding with STDP in a spiking recurrent neural network model of the hippocampus. PLoS Comput. Biol. *6*, e1000839.

Bush, D., Philippides, A., Husbands, P., and O'Shea, M. (2010b). Spike-timing dependent plasticity and the cognitive map. Front. Comput. Neurosci. *4*, 142.

Buzsáki, G. (1989). Two-stage model of memory trace formation: a role for "noisy" brain states. Neuroscience *31*, 551–570.

Buzsáki, G., Haas, H.L., and Anderson, E.G. (1987). Long-term potentiation induced by physiologically relevant stimulus patterns. Brain Res. *435*, 331–333.

Carlisle, H.J., Fink, A.E., Grant, S.G., and O'Dell, T.J. (2008). Opposing effects of PSD-93 and PSD-95 on long-term potentiation and spike timing-dependent plasticity. J. Physiol. *586*, 5885–5900.

Carr, M.F., Jadhav, S.P., and Frank, L.M. (2011). Hippocampal replay in the awake state: a potential substrate for memory consolidation and retrieval. Nat. Neurosci. *14*, 147–153.

Chauvette, S., Seigneur, J., and Timofeev, I. (2012). Sleep oscillations in the thalamocortical system induce long-term neuronal plasticity. Neuron *75*, 1105–1113.

Clopath, C., Büsing, L., Vasilaki, E., and Gerstner, W. (2010). Connectivity reflects coding: a model of voltage-based STDP with homeostasis. Nat. Neurosci. *13*, 344–352.

Davidson, T.J., Kloosterman, F., and Wilson, M.A. (2009). Hippocampal replay of extended experience. Neuron *63*, 497–507.

Debanne, D., Gähwiler, B.H., and Thompson, S.M. (1998). Long-term synaptic plasticity between pairs of individual CA3 pyramidal cells in rat hippocampal slice cultures. J. Physiol. *507*, 237–247.

Diba, K., and Buzsáki, G. (2007). Forward and reverse hippocampal place-cell sequences during ripples. Nat. Neurosci. *10*, 1241–1242.

Ego-Stengel, V., and Wilson, M.A. (2010). Disruption of ripple-associated hippocampal activity during rest impairs spatial learning in the rat. Hippocampus *20*, 1–10.

Faber, E.S., Delaney, A.J., and Sah, P. (2005). SK channels regulate excitatory synaptic transmission and plasticity in the lateral amygdala. Nat. Neurosci. *8*, 635–641.

Foster, D.J., and Wilson, M.A. (2006). Reverse replay of behavioural sequences in hippocampal place cells during the awake state. Nature *440*, 680–683.

Frankland, P.W., and Bontempi, B. (2005). The organization of recent and remote memories. Nat. Rev. Neurosci. *6*, 119–130.

Gais, S., Lucas, B., and Born, J. (2006). Sleep after learning aids memory recall. Learn. Mem. *13*, 259–262.

Gambino, F., Pagès, S., Kehayas, V., Baptista, D., Tatti, R., Carleton, A., and Holtmaat, A. (2014). Sensory-evoked LTP driven by dendritic plateau potentials in vivo. Nature *515*, 116–119.

Girardeau, G., Benchenane, K., Wiener, S.I., Buzsáki, G., and Zugaro, M.B. (2009). Selective suppression of hippocampal ripples impairs spatial memory. Nat. Neurosci. *12*, 1222–1223.

Golding, N.L., Staff, N.P., and Spruston, N. (2002). Dendritic spikes as a mechanism for cooperative long-term potentiation. Nature *418*, 326–331.

Groen, M.R., Paulsen, O., Pérez-Garci, E., Nevian, T., Wortel, J., Dekker, M.P., Mansvelder, H.D., van Ooyen, A., and Meredith, R.M. (2014). Development of dendritic tonic GABAergic inhibition regulates excitability and plasticity in CA1 pyramidal neurons. J. Neurophysiol. *112*, 287–299.

Grosmark, A.D., Mizuseki, K., Pastalkova, E., Diba, K., and Buzsáki, G. (2012). REM sleep reorganizes hippocampal excitability. Neuron *75*, 1001–1007.

Hebb, D. (1949). The Organisation of Behaviour (Wiley).

Isaac, J.T., Buchanan, K.A., Muller, R.U., and Mellor, J.R. (2009). Hippocampal place cell firing patterns can induce long-term synaptic plasticity in vitro. J. Neurosci. *29*, 6840–6850.

Jadhav, S.P., Kemere, C., German, P.W., and Frank, L.M. (2012). Awake hippocampal sharp-wave ripples support spatial memory. Science *336*, 1454–1458.

Karlsson, M.P., and Frank, L.M. (2009). Awake replay of remote experiences in the hippocampus. Nat. Neurosci. *12*, 913–918.

King, C., Henze, D.A., Leinekugel, X., and Buzsáki, G. (1999). Hebbian modification of a hippocampal population pattern in the rat. J. Physiol. *521*, 159–167.

Klausberger, T., Magill, P.J., Márton, L.F., Roberts, J.D., Cobden, P.M., Buzsáki, G., and Somogyi, P. (2003). Brain-state- and cell-type-specific firing of hippocampal interneurons in vivo. Nature *421*, 844–848.

Kumar, A., and Mehta, M.R. (2011). Frequency-dependent changes in NMDAR-dependent synaptic plasticity. Front. Comput. Neurosci. *5*, 38.

Kwag, J., and Paulsen, O. (2009). The timing of external input controls the sign of plasticity at local synapses. Nat. Neurosci. *12*, 1219–1221.

Lee, A.K., and Wilson, M.A. (2002). Memory of sequential experience in the hippocampus during slow wave sleep. Neuron *36*, 1183–1194.

Li, X.G., Somogyi, P., Ylinen, A., and Buzsáki, G. (1994). The hippocampal CA3 network: an in vivo intracellular labeling study. J. Comp. Neurol. *339*, 181–208.

Louie, K., and Wilson, M.A. (2001). Temporally structured replay of awake hippocampal ensemble activity during rapid eye movement sleep. Neuron *29*, 145–156.

Magee, J.C., and Johnston, D. (1997). A synaptically controlled, associative signal for Hebbian plasticity in hippocampal neurons. Science *275*, 209–213.

Maier, N., Tejero-Cantero, A., Dorrn, A.L., Winterer, J., Beed, P.S., Morris, G., Kempter, R., Poulet, J.F., Leibold, C., and Schmitz, D. (2011). Coherent phasic excitation during hippocampal ripples. Neuron *72*, 137–152.

Maret, S., Faraguna, U., Nelson, A.B., Cirelli, C., and Tononi, G. (2011). Sleep and waking modulate spine turnover in the adolescent mouse cortex. Nat. Neurosci. *14*, 1418–1420.

Marshall, L., and Born, J. (2007). The contribution of sleep to hippocampus-dependent memory consolidation. Trends Cogn. Sci. *11*, 442–450.

Mehta, M.R., Quirk, M.C., and Wilson, M.A. (2000). Experience-dependent asymmetric shape of hippocampal receptive fields. Neuron *25*, 707–715.

Min, R., and Nevian, T. (2012). Astrocyte signaling controls spike timing-dependent depression at neocortical synapses. Nat. Neurosci. *15*, 746–753.

Morris, R.G.M., Anderson, E., Lynch, G.S., and Baudry, M. (1986). Selective impairment of learning and blockade of long-term potentiation by an N-methyl-D-aspartate receptor antagonist, AP5. Nature *319*, 774–776.

Muller, R.U., Kubie, J.L., and Ranck, J.B., Jr. (1987). Spatial firing patterns of hippocampal complex-spike cells in a fixed environment. J. Neurosci. *7*, 1935–1950.

Muller, R.U., Stead, M., and Pach, J. (1996). The hippocampus as a cognitive graph. J. Gen. Physiol. *107*, 663–694.

Nádasdy, Z., Hirase, H., Czurkó, A., Csicsvari, J., and Buzsáki, G. (1999). Replay and time compression of recurring spike sequences in the hippocampus. J. Neurosci. *19*, 9497–9507.

Nevian, T., and Sakmann, B. (2006). Spine Ca2+ signaling in spike-timing-dependent plasticity. J. Neurosci. *26*, 11001–11013.

Ngo-Anh, T.J., Bloodgood, B.L., Lin, M., Sabatini, B.L., Maylie, J., and Adelman, J.P. (2005). SK channels and NMDA receptors form a Ca2+-mediated feedback loop in dendritic spines. Nat. Neurosci. *8*, 642–649.

O'Connor, D.H., Wittenberg, G.M., and Wang, S.S. (2005). Graded bidirectional synaptic plasticity is composed of switch-like unitary events. Proc. Natl. Acad. Sci. USA *102*, 9679–9684.

O'Keefe, J., and Dostrovsky, J. (1971). The hippocampus as a spatial map. Preliminary evidence from unit activity in the freely-moving rat. Brain Res. *34*, 171–175.

O'Neill, J., Senior, T.J., Allen, K., Huxter, J.R., and Csicsvari, J. (2008). Reactivation of experience-dependent cell assembly patterns in the hippocampus. Nat. Neurosci. *11*, 209–215.

O'Neill, J., Pleydell-Bouverie, B., Dupret, D., and Csicsvari, J. (2010). Play it again: reactivation of waking experience and memory. Trends Neurosci. *33*, 220–229.

Pavlides, C., and Winson, J. (1989). Influences of hippocampal place cell firing in the awake state on the activity of these cells during subsequent sleep episodes. J. Neurosci. *9*, 2907–2918.

Pike, F.G., Meredith, R.M., Olding, A.W.A., and Paulsen, O. (1999). Rapid report: postsynaptic bursting is essential for 'Hebbian' induction of associative long-term potentiation at excitatory synapses in rat hippocampus. J. Physiol. *518*, 571–576.

Rackham, O.J., Tsaneva-Atanasova, K., Ganesh, A., and Mellor, J.R. (2010). A Ca-based computational model for NMDA receptor-dependent synaptic plasticity at individual post-synaptic spines in the hippocampus. Front. Synaptic Neurosci. *2*, 31.

Rodríguez-Moreno, A., and Paulsen, O. (2008). Spike timing-dependent long-term depression requires presynaptic NMDA receptors. Nat. Neurosci. *11*, 744–745.

Rosanova, M., and Ulrich, D. (2005). Pattern-specific associative long-term potentiation induced by a sleep spindle-related spike train. J. Neurosci. *25*, 9398–9405.

Rudoy, J.D., Voss, J.L., Westerberg, C.E., and Paller, K.A. (2009). Strengthening individual memories by reactivating them during sleep. Science *326*, 1079.

Sadowski, J.H., Jones, M.W., and Mellor, J.R. (2011). Ripples make waves: binding structured activity and plasticity in hippocampal networks. Neural Plast. *2011*, 960389.

Sheffield, M.E., and Dombeck, D.A. (2015). Calcium transient prevalence across the dendritic arbour predicts place field properties. Nature *517*, 200–204.

Shouval, H.Z., Bear, M.F., and Cooper, L.N. (2002). A unified model of NMDA receptor-dependent bidirectional synaptic plasticity. Proc. Natl. Acad. Sci. USA *99*, 10831–10836.

Sjöström, P.J., Turrigiano, G.G., and Nelson, S.B. (2001). Rate, timing, and cooperativity jointly determine cortical synaptic plasticity. Neuron *32*, 1149–1164.

Sjöström, P.J., Turrigiano, G.G., and Nelson, S.B. (2003). Neocortical LTD via coincident activation of presynaptic NMDA and cannabinoid receptors. Neuron *39*, 641–654.

Skaggs, W.E., McNaughton, B.L., Wilson, M.A., and Barnes, C.A. (1996). Theta phase precession in hippocampal neuronal populations and the compression of temporal sequences. Hippocampus *6*, 149–172.

Tigaret, C.M., Olivo, V., Sadowski, J.H.L.P., Ashby, M.C., and Mellor, J.R. (2016). Coordinated activation of distinct Ca(2+) sources and metabotropic glutamate receptors encodes Hebbian synaptic plasticity. Nat. Commun. *7*, 10289.

Tononi, G., and Cirelli, C. (2014). Sleep and the price of plasticity: from synaptic and cellular homeostasis to memory consolidation and integration. Neuron *81*, 12–34.

Varga, C., Golshani, P., and Soltesz, I. (2012). Frequency-invariant temporal ordering of interneuronal discharges during hippocampal oscillations in awake mice. Proc. Natl. Acad. Sci. USA *109*, E2726–E2734.

Vyazovskiy, V.V., Cirelli, C., Pfister-Genskow, M., Faraguna, U., and Tononi, G. (2008). Molecular and electrophysiological evidence for net synaptic potentiation in wake and depression in sleep. Nat. Neurosci. *11*, 200–208.

Whitlock, J.R., Heynen, A.J., Shuler, M.G., and Bear, M.F. (2006). Learning induces long-term potentiation in the hippocampus. Science *313*, 1093–1097.

Williams, S.R., and Mitchell, S.J. (2008). Direct measurement of somatic voltage clamp errors in central neurons. Nat. Neurosci. *11*, 790–798.

Williams, S.R., Wozny, C., and Mitchell, S.J. (2007). The back and forth of dendritic plasticity. Neuron *56*, 947–953.

Wilson, M.A., and McNaughton, B.L. (1994). Reactivation of hippocampal ensemble memories during sleep. Science *265*, 676–679.

Wittenberg, G.M., and Wang, S.S.H. (2006). Malleability of spike-timing-dependent plasticity at the CA3-CA1 synapse. J. Neurosci. *26*, 6610–6617.

Cell Reports, Volume *14*

Supplemental Information

Sharp-Wave Ripples Orchestrate the Induction of Synaptic Plasticity during Reactivation of Place Cell Firing Patterns in the Hippocampus

Josef H.L.P. Sadowski, Matthew W. Jones, and Jack R. Mellor

Supplementary Figure 1

a

b

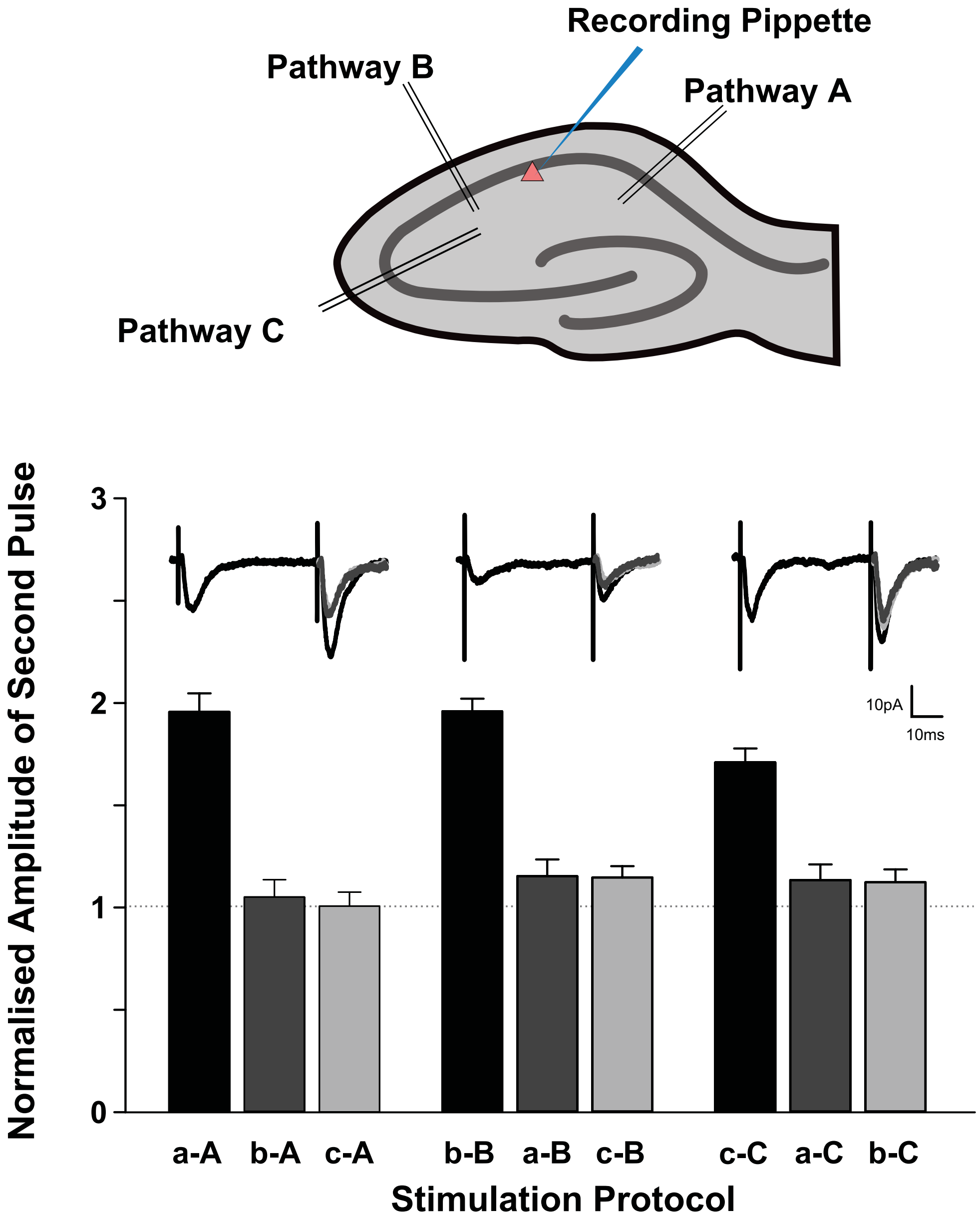

Supplemental Figure S1. Three-way Schaffer collateral pathway separation in stratum radiatum, related to Figure 2.

a) Three bipolar stimulating electrodes were positioned in stratum radiatum, activating three independent input pathways to a postsynaptic target cell in CA1.

b) EPSCs in 7 sample slices were recorded in response to a sequence of paired pulse stimuli delivered across all three pathways. Pulses were separated by 50ms. Pulses to the same pathway produced robust paired pulse facilitation where as those delivered to two opposing pathways did not. Example traces shown above.
